# First Clinical Case Report of a Xenograft–Allograft Combination for Alveolar Ridge Augmentation Using a Bovine Bone Substitute Material with Hyaluronate (Cerabone^®^ Plus) Combined with Allogeneic Bone Granules (Maxgraft^®^)

**DOI:** 10.3390/jcm12196214

**Published:** 2023-09-26

**Authors:** Frank R. Kloss, Peer W. Kämmerer, Anita Kloss-Brandstätter

**Affiliations:** 1 Private Clinic for Oral- and Maxillofacial Surgery, Kärntnerstraße 62, 9900 Lienz, Austria; info@mkg-kloss.at; 2 Department of Oral and Maxillofacial Surgery, University Medical Center Mainz, Augustusplatz 2, 55131 Mainz, Germany; peer.kaemmerer@gmx.de; 3 Department of Engineering & IT, Carinthia University of Applied Sciences, Europastraße 4, 9524 Villach, Austria

**Keywords:** alveolar ridge augmentation, heterografts, allografts, dental implants, case reports

## Abstract

Background: A patient had lost the first left maxillary incisor in the esthetic zone. Methods: The defect in the alveolar ridge was reconstructed for an implant-supported restoration using a new xenogeneic bone substitute containing hyaluronate, which was used in combination with allogeneic bone granules. Results: After three years of follow-up, the dental implant was stable and showed no signs of infection. Conclusions: This is the first case report with a long-term follow-up time of three years of a successful clinical application of a xenograft–allograft combination (cerabone^®^ plus combined with maxgraft^®^) for alveolar ridge augmentation before dental implantation. Cerabone^®^ plus offers volume stability, provides reliable and efficient structural support of the oral soft tissues in the augmented region (particularly crucial in the aesthetic zone), and preserves the alveolar ridge shape.

## 1. Background

Alveolar ridge augmentation is vital for dental implant success since it helps in restoring bone structure in the jaw. Allogeneic (human donor) and xenogeneic (animal source) bone substitutes, besides others, offer a safe and effective way to enhance bone volume, ensuring stable and long-lasting dental implants, and omit the need of autograft harvesting [1,2,3]. Advancements in medical technology have led to remarkable developments in the field of bone grafting, improving the outcomes of various oral surgical procedures [4,5,6,7,8]. Among these breakthroughs, cerabone^®^ plus has emerged as a promising bone graft material with exceptional handling properties when applied for alveolar ridge augmentation before dental implantation. In this product, the bovine bone graft cerabone^®^, premixed with hyaluronate (water-soluble salt form of hyaluronic acid), is provided to form cerabone^®^ plus. It transforms into a “sticky-bone” graft substitute when in contact with saline or blood, providing exceptional simplicity of use by aiding both uptake and administration into the defect area.

### 1.1. Bovine Bone Substitutes

Numerous studies have demonstrated that the bovine bone substitute cerabone^®^ is proven and suitable for reconstructive oral surgery followed by dental implantation [7,9,10,11,12,13,14,15]. The excellent hydrophilicity of cerabone^®^ has been demonstrated via high-speed microscopical image analysis [16]. In accordance, an analysis of the regeneration capacity and immune response to xenogeneic and synthetic bone substitute materials showed that the xenogeneic cerabone^®^ induced a higher anti-inflammatory reaction [17,18,19]. Accordingly, a histologic, histomorphometric, and radiographic analysis of cerabone^®^ used in sinus floor elevation found that this bovine bone substitute material proved to be effective in slowly resorbing osseoconductive material [20].

### 1.2. Biofunctionalization of Bone Substitute Materials

Since it has already been shown that cerabone^®^ has advantageous properties for oral bone regeneration, the question arises why further biofunctionalization is necessary. Screening of hydroxyapatite-containing biomaterials (including cerabone^®^, Bio-Oss^®^ and maxresorb^®^) for alveolar ridge augmentation using an animal model found that none of the hydroxyapatite biomaterials encouraged local bone production or maturation beyond this animal model’s innate regenerating capacity, indicating their limits as a regenerative tool [21]. To improve bony healing and thus the integration and stability of the dental implants, cerabone^®^ was combined with platelet-rich fibrin (PRF). Indeed, the combination of naturally derived bovine hydroxyapatite (cerabone^®^) with PRF provided additional growth factors which enhanced the wound healing process and maintained the volumes [22]. Unfortunately, preparing platelet concentrates such as PRF involves drawing blood from the patient. Therefore, it was considered whether biofunctionalization could also be achieved with hyaluronic acid instead of PRF [23,24].

Hyaluronic acid is an essential component of the human body’s tissues and organs, and is crucial for numerous physiological processes such as cell migration, wound healing, and tissue hydration. Recently, improved bioactive polymers for bone regeneration have been extensively studied, including hyaluronic acid [25]. Because this natural polymer is nontoxic, noninflammatory, biodegradable, and biocompatible, many medical devices containing hyaluronic acid have been developed. Additionally, hyaluronic acid-based composite scaffolds have demonstrated strong promise for fostering osteogenesis and mineralization [26]. Improved osteogenesis and osseointegration can be achieved with hyaluronic acid-based microparticles that covalently attach to metal implant surfaces and release bioactive components [25]. The advantage of biofunctionalization of the hydroxyapatites with hyaluronic acid results from the fact that hyaluronic acid is a hydrophilic molecule whose salt form (hyaluronate) can be activated easily by adding saline solution, thus replacing the need to draw blood from the patient.

In vitro studies showed that hyaluronic acid biofunctionalization of the bovine bone substitute cerabone^®^ caused an increased activity of human osteoblasts, thus indicating the ability to accelerate and improve bone regeneration [24,27]. Based on the findings of the in vitro studies, the innovative bone graft substitute was tested in in vivo models. The efficacy of bone substitute materials for maxillofacial osseous regeneration is closely correlated with the efficient induction of angiogenesis. In vivo angiogenesis was significantly stimulated when hyaluronic acid was combined with the xenogeneic bone substitute material cerabone^®^ [23]. In further animal models, cerabone^®^ plus provided excellent biocompatibility and good osteoconductive properties [28,29]. In addition, cerabone^®^ plus was successfully used in peri-implantitis reconstructive surgery [30] and in one clinical case for alveolar ridge augmentation [31].

### 1.3. Clinical Applications

Cerabone^®^ plus received CE certification in 2020 (https://botiss.com/de/botiss-entwickelt-innovatives-knochenersatzmaterial-mit-hyaluronsaeure/ (accessed on 11 August 2023)). Given the properties of cerabone^®^ plus, including osteoconductivity and volume stability, this relatively new bovine bone substitute material enables time-saving and efficient defect filling and easy defect contouring [30,31]. After hydration, cerabone^®^ plus is sticky and malleable, thereby minimizing displacement of single granules during application [24,27]. It can therefore be applied for horizontal and vertical augmentation [31], peri-implant defects [30], socket preservation and sinus lifts. 

This is the first case report of a clinical application of cerabone^®^ plus in combination with allogeneic bone granules for alveolar ridge augmentation followed by dental implantation with a follow-up of three years.

## 2. Materials and Methods

### 2.1. Overview of the Clinical Case

**Figure 1:**
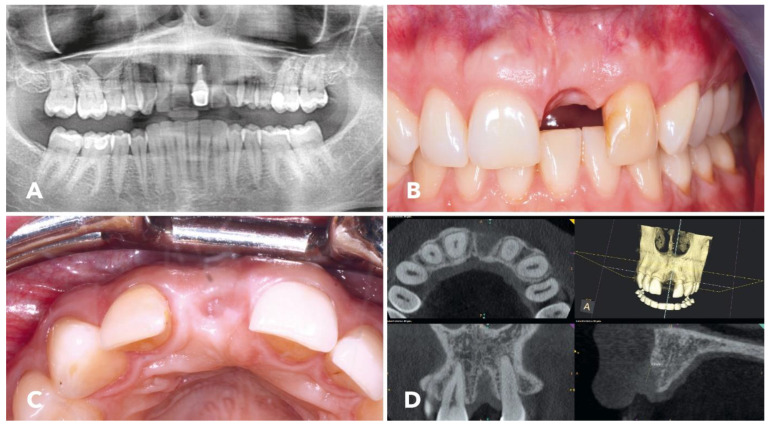
(**A**) Preoperative panoramic radiograph showing a periradicular translucent lesion on tooth 21; clinically, a buccal fistula was present. (**B**) Clinical situation of the patient 4 months after the extraction of tooth 21; the distal papilla had receded and the buccal wall 21 was atrophic; the soft tissue situation was sound and free of any inflammation; a wide zone of keratinized gingiva was visible. (**C**) Clinical situation in the palatinal view; a slight lack of buccal bone was evident; no signs of inflammation. (**D**) The cone beam CT showed the transverse bone defect, which corresponded to a class II defect according to the ITI classification.

This is a retrospective case report, completed according to the CARE guidelines [32,33]. A 34-year-old female patient presented with the wish for a fixed prosthetic rehabilitation at site 21. The first left maxillary incisor suffered from a trauma 15 years ago and was mobile. A root resection had been performed twice. Figure 1A shows a panoramic radiograph demonstrating a periradicular lesion in the region of tooth 21. A clinical examination revealed the presence of a buccal fistula. Therefore, the tooth was extracted. Figure 1B depicts the clinical situation of the patient four months after the extraction of tooth 21. The distal papilla had receded, and the buccal wall at tooth 21 was atrophic. The soft tissue situation was sound and free of any inflammation. A broad zone of keratinized gingiva was visible. Figure 1C shows the clinical situation in a palatinal view. A slight lack of buccal bone was evident, but no signs of inflammation were detected.

Preoperatively a cone beam CT, which was taken immediately before augmentative surgery (Figure 1D), showed a transverse bone defect of the alveolar ridge with a residual ridge width of 2–3 mm, resembling a Type-II defect according to Chen and Buser [34], or a Class 4 defect (horizontal ridge defect requiring bone augmentation before implant placement) according to Benic and Hämmerle [35]. According to the ITI recommendation for reconstruction of the osseous deficit, a two-stage GBR (Guided Bone Regeneration) procedure was planned. The safety assessment code matrix for the patient according to Dawson and Chen [36] can be found in Table 1. The patient was healthy without any symptoms of periodontitis. No medication on a regular basis was necessary.

This osseous defect was restored with cerabone^®^ plus (botiss biomaterials GmbH, Zossen, Germany); a pure bovine bone mineral with hyaluronate added, which was used in combination with allogeneic bone granules (maxgraft^®^, botiss biomaterials GmbH, Zossen, Germany). A titanium implant was inserted six months after augmentation. The last control picture was taken in June 2023. The total follow-up time after augmentation was approximately 3 years. The intervention was conducted under local anesthesia in our private practice in Lienz, Austria.

### 2.2. Cerabone^®^ Plus

**Figure 2:**
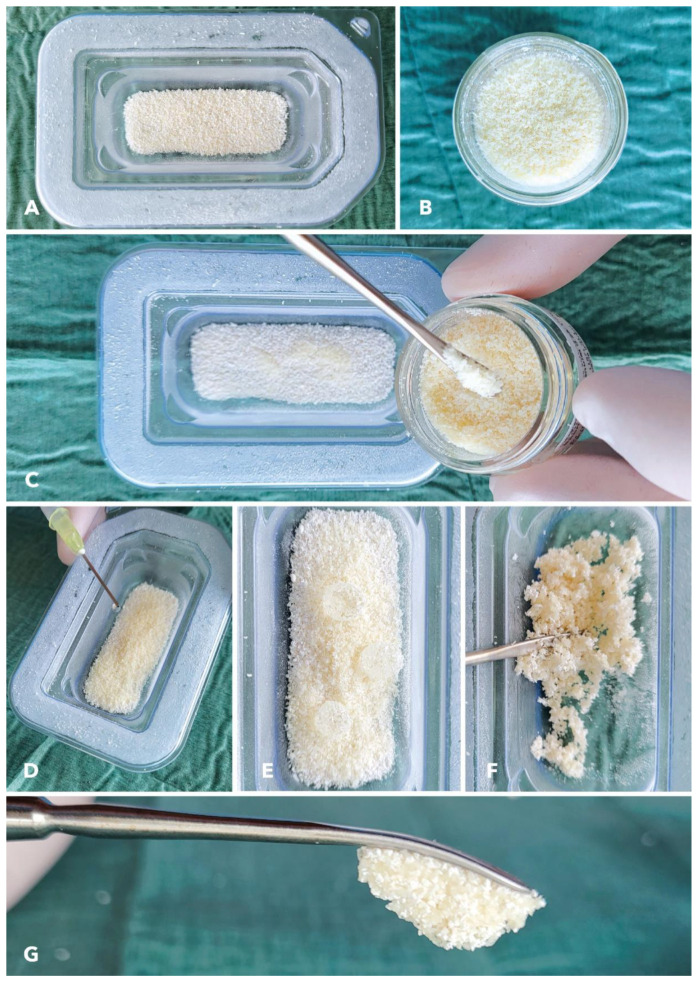
Handling of cerabone^®^ plus. (**A**) Blister containing cerabone^®^ plus after opening. (**B**) Allograft bone substitute maxgraft^®^ cancellous granules. (**C**) Addition of maxgraft^®^ granules to cerabone^®^ plus to increase the cohesiveness. (**D**) Hydration of the xenograft/allograft mix in the provided blister using sterile saline solution. (**E**) Only very little liquid is needed for graft hydration thanks to the water-binding capacity of the hyaluronate contained in cerabone^®^ plus. (**F**) Thorough mixing of the added liquid with the biomaterials mix. (**G**) Ready-to-use sticky bone.

**Figure 3:**
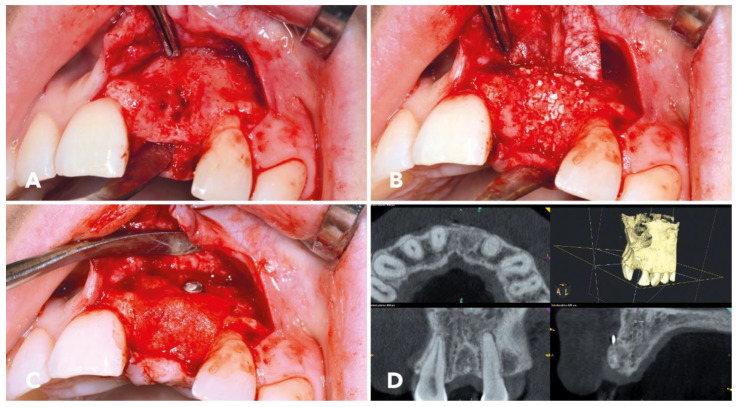
(**A**) Intraoperative situs showing the class II defect; the intraosseous compartment was irregularly configured. (**B**) The collagenous Jason^®^ membrane is already in situ; the cerabone^®^ plus in combination with the maxgraft^®^ granules appeared to be compact and firmly bonded to the underlying bone. (**C**) Jason^®^ membrane covering the augmented area; the pin fixed the membrane buccally; palatally, the membrane was placed under the palatal gingiva. (**D**) Cone beam CT scan 4 months after augmentation. The defect was already partially regenerated; the buccal volume, especially in the crestal region, was reconstructed and preserved.

Cerabone^®^ plus (botiss biomaterials GmbH, Zossen, Germany) is a combination of the established bovine bone grafting material cerabone^®^ and sodium hyaluronate, which is a water-soluble form of hyaluronic acid. The cerabone^®^ granules are delivered with hyaluronate in a container for convenient hydration (Figure 2). Due to the pronounced liquid binding capacities of hyaluronate, cerabone^®^ plus forms a sticky bone material upon hydration that provides unique application comfort by allowing both easy uptake and delivery to application site. It possesses excellent biocompatibility and osteoconductivity, making it an ideal substitute for the patient’s own bone when it comes to alveolar ridge augmentation procedures [23,24]. By adding allogeneic granules to the bovine bone, the regenerated tissue becomes more homogenous and less dense (postoperative CBCT Figure 3D).

### 2.3. Preparation and Handling of Cerabone^®^ Plus in Combination with Maxgraft^®^

Cerabone^®^ plus is supplied in sterile blisters and contains the number of granules needed for augmentation before placing a single implant (Figure 2). In the first step, maxgraft^®^ cancellous granules, an allograft bone substitute material (botiss biomaterials GmbH, Zossen, Germany) that has been demonstrated to enhance bone formation and exhibit rapid turnover into vital bone, are added to the cerabone^®^ plus granules to increase the cohesiveness. Then, the xenograft/allograft mix is hydrated in the provided blister using sterile saline solution. Very less amounts of liquid is needed for graft hydration, due to the water-binding activities of the hyaluronate contained in cerabone^®^ plus. After a thorough mixing of the added liquid with the biomaterials mix, a ready-to-use sticky bone is present. While defects within the bony envelope might benefit from a higher ratio of allografts, defects outside of the contour or in the esthetic zone might benefit from a higher ratio of the resorption stable cerabone^®^. The more maxgraft^®^ granules are added, the firmer the consistency becomes. The result is a highly malleable mass in the sense of a sticky bone that easily adapts to the defect.

### 2.4. Surgical Procedure

The pre-operative clinical examinations, the overall surgical procedure and the post-operative care followed our previous descriptions of alveolar ridge augmentation [1,4,5]. 

After opening the surgical site, a class II osseous defect was revealed (Figure 3A). The intraosseous compartment was irregularly configured. The defect was reconstructed with the help of the “sticky xeno-allograft bone” (cerabone^®^ plus with maxgraft^®^ granules). Care was taken to use the contour of the surrounding intact alveolar bone to guide the reconstruction. Figure 3B illustrates the situation after sticky xeno-allograft bone had been placed in the defect area. The xenogeneic–allogeneic bone substitute mixture appeared to be compact and firmly bonded to the underlying bone. The surgical site was protected with a slowly resorbing barrier membrane made of porcine pericardium (Jason^®^ membrane, botiss biomaterials GmbH, Zossen, Germany). The membrane was fixed with a titanium pin buccally; palatally, it was placed under the periosteum. Figure 3C shows the collagen membrane in situ and fixed over the graft.

A cone-beam computed tomography was taken four months after the augmentation (Figure 3D), demonstrating that the defect was already partially regenerated. The buccal volume had been reconstructed and preserved, especially in the crestal region.

Routine postoperative care included the administration of amoxicillin and clavulanic acid (625 mg, administered orally, three times a day for four days), ibuprofen (600 mg, administered orally, every six hours as needed), and mouthwashes (0.2% chlorhexidine, three times daily for seven days). The patient was recalled at monthly intervals for a period of six months to detect possible complications, such as infection, pain, discomfort, graft exposure, and mobility. 

### 2.5. Implantation

**Figure 4:**
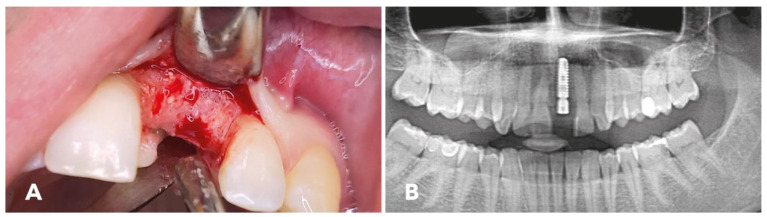
(**A**) Reconstructed alveolar ridge at re-entry 6 months after augmentation surgery; cerabone^®^ granules surrounded by bleeding bone are visible superficially. (**B**) Postoperative panoramic radiograph with inserted implant.

Six months post surgery, reopening (Figure 4A) and implantation were performed. The bleeding bone indicated satisfactory osseous transformation and good blood supply to the graft. The implant was placed as described previously [1]. The membrane fixation pin was removed, and graft stability was assessed. The bone substitute was found to be well integrated. A satisfactory bone volume for stable implantation was achieved. Subsequently, the patient received a titanium implant in site 21 (Medentika; Straumann Group, Basel, Switzerland). Figure 4B illustrates the stable positioning of the implant in region 21. The implant was uncovered three months after implantation, and the patient received a screw-retained crown.

## 3. Results

There were no signs of infection, wound dehiscence, graft exposure or other postoperative complications during the healing period following bone augmentation. At the time of implantation, the mix of xeno and allograft was well integrated into the recipient site, and the formerly lacking bone volume was sufficiently restored. The implant was stable during follow-up.

No bleeding on probing was recognizable at six measuring points per implant. There was no evidence of mucositis or periimplantitis.

**Figure 5:**
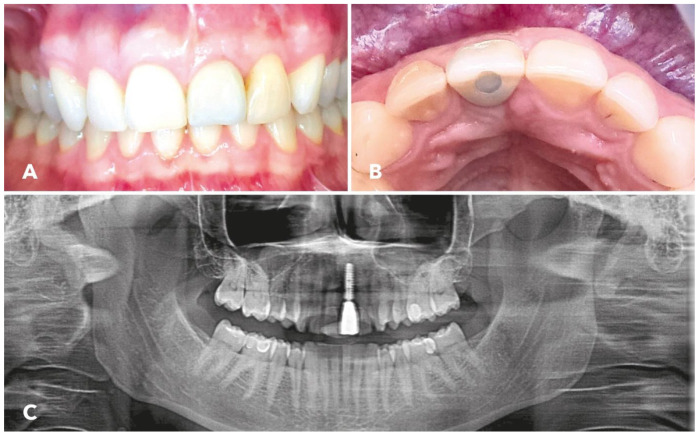
(**A**) Frontal view 1 year after the prosthetic restoration of implant 21; the color of crown 21 is slightly darker compared to 11; the papilla distal to implant 21 has been reconstructed. (**B**) View after prosthetic rehabilitation with slight defect of the buccal outer contour. (**C**) Panoramic radiograph 3 years after prosthetic rehabilitation.

Figure 5A,B shows the final situation 12 months after the definitive restoration. 

Three years postoperatively the soft tissue situation was stable without signs of mucositis at the implant site. The panoramic radiograph, which was taken three years after augmentation (Figure 5C), also revealed excellent osseointegration of the implant. No signs of bone loss at the implant were detectable.

## 4. Discussion

Cerabone^®^ plus represents a significant advancement in bone grafting materials, offering remarkable regenerative potential, angiogenic properties, and mechanical stability. Cerabone^®^ plus has emerged as a versatile and reliable option for a wide range of applications in oral surgery [23,24,27,28,29,30,31]. In contrast to traditional granular bone graft substitutes, cerabone^®^ plus creates a cohesive mass after being hydrated because of the prominent liquid-binding ability of hyaluronate. Cerabone^®^ plus makes defect augmentation effective and permits precise application of the granules, as well as quick defect contouring.

### 4.1. Alveolar Ridge Augmentation

Alveolar ridge augmentation is a crucial pre-implantation procedure in dentistry that aims to enhance the bone structure of the jaw to provide a solid foundation for dental implants [37,38]. When a tooth is lost or extracted, the surrounding alveolar bone will undergo resorption, leading to a decrease in bone volume and density [39,40,41]. Without sufficient bone support, dental implants may fail to integrate properly, resulting in compromised aesthetics and function [42,43].

The process of alveolar ridge augmentation involves grafting bone material into the depleted area to stimulate new bone formation or provide support for dental implants [38]. To this end, autologous micrografts have shown safety and predictable results [44]. Among the various bone graft options, allogeneic and xenogeneic bone substitutes play a vital role due to their unique advantages [6,45].

Allogeneic bone substitutes are derived from human donors [46]. Allografts such as demineralized freeze-dried bone allograft or mineralized processed bone allograft, undergo rigorous processing and sterilization to ensure safety and minimize immune reactions [47]. The availability of allogeneic grafts is abundant, making them a practical choice for dental surgeons [48]. Moreover, they possess osteoconductive properties, meaning they provide a scaffold for bone growth, promoting fast natural bone regeneration [49,50]. Several studies have demonstrated no differences in implant success, bone resorption and complication rates after horizontal ridge augmentation in allogeneic as compared to autogenous bone blocks [1,4,51].

On the other hand, xenogeneic bone substitutes are sourced from animals, usually bovine or porcine sources [45,52,53]. Like allogeneic grafts, they are processed and purified to minimize the risk of disease transmission and immunogenic reactions [7,54]. Xenogeneic bone substitutes also offer excellent osteoconductive properties, and have been widely used in dental implant procedures [7,9,10,11,12,13,18,19]. In addition, high-temperature-treated bovine bone substitute materials such as cerabone^®^ provide long-term volume stability due to slow resorption [11].

### 4.2. Enhanced Bone Regeneration

Several studies consistently demonstrated the regenerative potential of cerabone^®^ plus, and its ability to support new bone formation [23,24,27,28,29,30,31]. Cerabone^®^ plus exhibited excellent osteoconductivity and biocompatibility, facilitating robust bone formation and integration with the host tissue [24]. One study concluded that cerabone^®^ plus could serve as a viable alternative to autogenous bone grafts in various clinical scenarios [24]. Two different histomorphometrical studies by Pröhl et al. (2021) and Alkildani et al. (2023) in Wistar rats demonstrated that cerabone^®^ plus provided excellent biocompatibility and osteoconductive properties [28,29]. 

### 4.3. Promotion of Angiogenesis

Angiogenesis plays a critical role in bone regeneration by facilitating the growth of new blood vessels necessary for nutrient supply and waste removal. In this regard, cerabone^®^ plus has shown promising results. A study by Kyyak et al. (2022) reported that cerabone^®^ plus actively promoted angiogenesis, thereby supporting the development of a well-vascularized bone tissue environment [23]. These findings highlight the potential of cerabone^®^ plus to enhance the healing process and contribute to improved patient outcomes.

### 4.4. Enhanced Mechanical Properties

In addition to its regenerative properties, cerabone^®^ plus also exhibits enhanced mechanical properties, rendering it suitable for load-bearing applications. A study by Rakasevic et al. (2023) demonstrated that cerabone^®^ plus possessed favorable compressive strength and elastic modulus, providing stability and structural support for stabilizing implants suffering from peri-implantitis [30]. This characteristic is particularly crucial in reconstructive procedures of the alveolar ridge, where the graft material must withstand mechanical stress and promote long-term implant success.

The importance of using allogeneic and xenogeneic bone substitutes lies in their effectiveness, biocompatibility, and the ability to enhance the alveolar ridge’s structure efficiently. By mixing the two materials, their favorable properties are combined (volume stability from the bovine bone and fast regeneration from the allograft) [55]. By providing a stable base for dental implants, these bone substitutes emerge as a reliable option for implantation procedures [7,56]. Additionally, their availability and ease of use make them valuable options, especially when patients have limited autogenous bone for grafting. 

### 4.5. Comparison to Previous Studies

This clinical case report is the first to reflect on the long-term outcome of augmentation of the alveolar ridge using a combination of cerabone^®^ plus and maxgraft^®^; it also simultaneously describes a precise surgical approach. So far, only one study has been published that used a mixture of cerabone^®^ and maxgraft^®^ for treating a patient with a previous implant failure [55]. However, the researchers did not use cerabone^®^ plus.

There are already a few studies on cerabone plus that have appeared since the CE certification of this new bone graft substitute in 2020. On one hand, there are animal studies, both in vitro [24] and in vivo [23,28,29]. A clinical study that used cerabone^®^ plus for ridge reconstruction in patients suffering from peri-implantitis for the first time [30], and one clinical case for alveolar ridge augmentation [31] have been published.

But the present study shows the first clinical application of a xenograft–allograft combination for ridge augmentation with a follow-up of 3 years. Furthermore, the present study is particularly interesting because the bovine bone graft substitute cerabone^®^ plus was mixed with an allogeneic bone graft substitute, whereas the product description of cerabone^®^ plus recommends a mixture with autogenous bone. Thus, a new application possibility was demonstrated here, which completely saves the patient from having to harvest his own donor bone. This is progressive and trend-setting.

## 5. Conclusions

This is the first case report with a long-term follow-up time of three years showing a successful clinical application of a xenograft–allograft combination (cerabone^®^ plus combined with maxgraft^®^) for alveolar ridge augmentation before dental implantation. Cerabone^®^ plus offers volume stability, provides reliable and efficient structural support for the oral soft tissues in the augmented region (particularly crucial in the aesthetic zone), and preserves the alveolar ridge shape. It also functions as a resorption protection when combined with allogeneic or autologous bone.

## Figures and Tables

**Table 1:** Safety assessment code matrix of the patient.

	Low Risk	Medium Risk	High Risk
**Health status**	Healthy, cooperative and without immunological restriction		
**Tobacco use**	Non-smoker	1-10/d	>10/d
**Patient’s claim**	Low	Medium	High
**Height of the smile line**	Low	Medium	High
**Gingival biotype**	Fabric strong, flat garland shape	Medium strong, average garland shape	Fabric weak, steep garland shape
**Dental form type**	Rectangular		Triangular
**Local infection**	None	Chronic	Acute
**Bone level at neighboring teeth**	≤5 mm from the contact point	5.5–6.5 mm from the contact point	≥7 mm from the contact point
**Restoration status of neighboring teeth**	Untouched		Restored
**Width of the gap**	1 tooth (≥7 mm)	1 tooth (≤7 mm)	≥2 teeth
**Soft tissue condition**	Intact	Reduced	Defective
**Bone volume**	No defect	Horizontal defect	Vertical defect

Colors indicate the risk status of each item studied in the table.

## Data Availability

Not applicable.
